# Extracellular Matrix Remodeling by Fibroblast-MMP14 Regulates Melanoma Growth

**DOI:** 10.3390/ijms222212276

**Published:** 2021-11-12

**Authors:** Elke Pach, Maike Kümper, Julia E. Fromme, Jan Zamek, Fabian Metzen, Manuel Koch, Cornelia Mauch, Paola Zigrino

**Affiliations:** 1 Department of Dermatology and Venereology, Faculty of Medicine and University Hospital Cologne, University of Cologne, 50937 Cologne, Germany; elke.pach@uk-koeln.de (E.P.); maike.kuemper@uk-koeln.de (M.K.); julia.fromme@uk-koeln.de (J.E.F.); jan.zamek@uk-koeln.de (J.Z.); cornelia.mauch@uk-koeln.de (C.M.); 2 Mildred Scheel School of Oncology Aachen Bonn Cologne Düsseldorf (MSSO ABCD), 50937 Cologne, Germany; 3 Faculty of Medicine and University Hospital, Institute for Dental Research and Oral Musculoskeletal Biology and Center for Biochemistry, University of Cologne, 50937 Cologne, Germany; fabian.metzen@uk-koeln.de (F.M.); manuel.koch@uni-koeln.de (M.K.)

**Keywords:** MMP14, proteases, melanoma, collagen, extracellular matrix

## Abstract

Maintaining a balanced state in remodeling the extracellular matrix is crucial for tissue homeostasis, and this process is altered during skin cancer progression. In melanoma, several proteolytic enzymes are expressed in a time and compartmentalized manner to support tumor progression by generating a permissive environment. One of these proteases is the matrix metalloproteinase 14 (MMP14). We could previously show that deletion of MMP14 in dermal fibroblasts results in the generation of a fibrotic-like skin in which melanoma growth is impaired. That was primarily due to collagen I accumulation due to lack of the collagenolytic activity of MMP14. However, as well as collagen I processing, MMP14 can also process several extracellular matrices. We investigated extracellular matrix alterations occurring in the MMP14-deleted fibroblasts that can contribute to the modulation of melanoma growth. The matrix deposited by cultured MMP14-deleted fibroblast displayed an antiproliferative and anti-migratory effect on melanoma cells in vitro. Analysis of the secreted and deposited-decellularized fibroblast’s matrix identified a few altered proteins, among which the most significantly changed was collagen XIV. This collagen was increased because of post-translational events, while de novo synthesis was unchanged. Collagen XIV as a substrate was not pro-proliferative, pro-migratory, or adhesive, suggesting a negative regulatory role on melanoma cells. Consistent with that, increasing collagen XIV concentration in wild-type fibroblast-matrix led to reduced melanoma proliferation, migration, and adhesion. In support of its anti-tumor activity, enhanced accumulation of collagen XIV was detected in peritumoral areas of melanoma grown in mice with the fibroblast’s deletion of MMP14. In advanced human melanoma samples, we detected reduced expression of collagen XIV compared to benign nevi, which showed a robust expression of this molecule around melanocytic nests. This study shows that loss of fibroblast-MMP14 affects melanoma growth through altering the peritumoral extracellular matrix (ECM) composition, with collagen XIV being a modulator of melanoma progression and a new proteolytic substrate to MMP14.

## 1. Introduction

Matrix metalloproteinases (MMPs) are calcium-dependent, zinc-containing endopeptidases crucial for tissue remodeling during development and adulthood. They can degrade extracellular matrix (ECM) proteins and thus play an essential role in tissue turnover to maintain tissue homeostasis. Their dysregulation is associated with several pathological conditions such as fibrosis and cancer [1]. Based on their structure and substrate specificity, MMPs are classified into six major subgroups: collagenases, gelatinases, stromelysins, matrilysins, and membrane-type MMPs [2]. MMP14 was the first described membrane-anchored MMP, identified as a cellular receptor and activator for pro-MMP2 [3]. Besides its role in maintaining tissue homeostasis, MMP14 is up-regulated in many tumors and promotes their progression by regulating cellular processes such as proliferation, migration, and tumor cell invasion [4,5,6]. The essential role of MMP14 in tissue homeostasis and development has been emphasized in mice with overall MMP14 deletion, which suffer from severe defects in connective tissues, organs function, and bone development, all leading to early death [7,8,9]. To circumvent premature death and address the cell-specific role of MMP14 in vivo, we have previously used mice carrying a cell-specific MMP14 deletion [10,11,12,13]. Deleting MMP14 in dermal fibroblasts (MMP14^Sf−/−^) highlighted the critical collagenase function of MMP14 in skin homeostasis. Indeed, MMP14 deletion in fibroblasts led to the development of a fibrosis-like skin phenotype [13]. Dermal alterations, collagen I accumulation, and increased tissue stiffness also restrained melanoma growth [14]. In addition to collagen I, likely other collagens were altered around these tumors, as increased hydroxyproline would result from all types of collagens [14]. Collagens are a large superfamily of 28 different types presenting the most abundant proteins in the extracellular matrix of vertebrates. They are functional in maintaining tissue structure, stability, and mechanical properties [15,16] and are regulators of several cellular functions, including proliferation, apoptosis, differentiation, adhesion, and migration [17,18,19,20,21,22]. In the skin dermis, fibrillar type I and type III collagens represent the major collagens. However, types V, VI, XII, XIV, and XVI are also present in lower amounts [23].

We sought to identify the ECM molecules that are changed in the peritumoral tissue of MMP14^Sf−/−^ mice that contribute to altering melanoma growth. By investigating the matrix deposited by MMP14^Sf−/−^ and control fibroblast, we show that collagen XIV is enriched in the peritumoral tissue of MMP14^Sf−/−^ mice and has anti-tumor activity in melanoma cells.

## 2. Results

### 2.1. Negative Effect of MMP14^Sf−/−^ Fibroblast Matrix on Melanoma Proliferation and Migration

**Figure 1:**
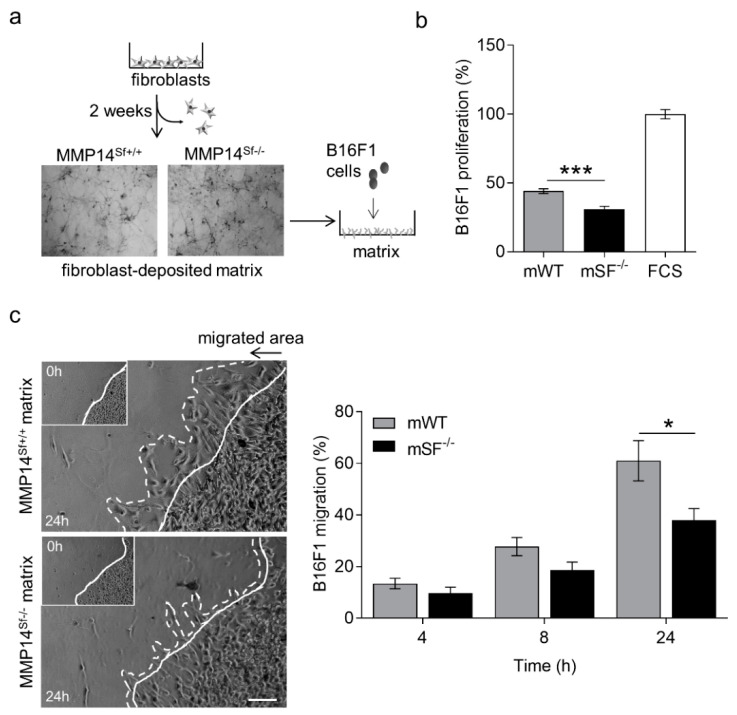
B16F1 cell proliferation and migration on MMP14^Sf−/−^ and MMP14^Sf+/+^ fibroblast matrix. (**a**) Schematic representation of fibroblast culture to obtain their matrix (picture of Coomassie-stained matrix after fibroblast removal) and seeding B16F1 cells on it. (**b**) BrdU incorporation measurement of B16F1 cells cultured on fibroblast matrix. (**c**) Colony outgrowth assay showing melanoma migration on fibroblast matrix (left) and quantification (right). Experiments were repeated three times. mWT (MMP14^Sf+/+^ matrix, control matrix) *n* = 3; mSF^−/−^ (MMP14^Sf−/−^ matrix) *n* = 3; continuous line, cell front after 0 h; dashed line, cell front after indicated period of time; * *p* < 0.05; *** *p* < 0.001; scale, 100 µm.

Extensive deposition of extracellular matrix, with enhanced collagen accumulation, is linked to the growth of many cancers [24,25,26], although the influence of collagen is diverse on the different types of cancers. Mice lacking MMP14 in stromal fibroblasts (MMP14^Sf−/−^) display skin fibrosis and reduced melanoma growth due to enhanced collagen I accumulation and increased skin stiffness [13,14]. However, it was unclear whether additional extracellular matrix alterations due to MMP14 deletion in fibroblasts, the primary producer of extracellular matrix in tissues, contribute to the observed effect. To address this, we cultured MMP14^Sf−/−^ and control fibroblasts for 14 days, the time necessary for cellular matrix deposition. As observed by Coomassie-stained wells after removing cells, the density of this matrix increased when cells lacked MMP14 (Figure 1a). The increase was quantified in urea-extracted matrices from 6 to 7 independent fibroblasts isolates and displayed a significant difference (** *p* < 0.01) with values of 16 ± 0.2 and 17.6 ± 0.3 µg/mL by control and MMP14^Sf−/−^ fibroblasts. Cell proliferation of three melanoma cell lines (B16F0, B16F1, and HCmel12) was significantly reduced when seeded on MMP14^Sf−/−^ fibroblast’s decellularized matrix compared to the control matrix and cells cultured on serum coating, used as an additional control (Figure 1b and Figure S1).

Further, B16F1 melanoma cells migration on matrix deposited from MMP14^Sf−/−^ and MMP14^Sf+/+^ fibroblasts for 24 h, analyzed by a colony outgrowth method, was decreased on MMP14^Sf−/−^ fibroblast matrix compared to control (Figure 1c). After four hours, this delay was detected and most significant at 24 h (Figure 1c). Collectively, these data indicate that matrix from MMP14^Sf−/−^ fibroblast negatively influences melanoma cell proliferation and migration.

### 2.2. Altered Composition of MMP14^Sf−/−^ Fibroblast ECM

**Figure 2:**
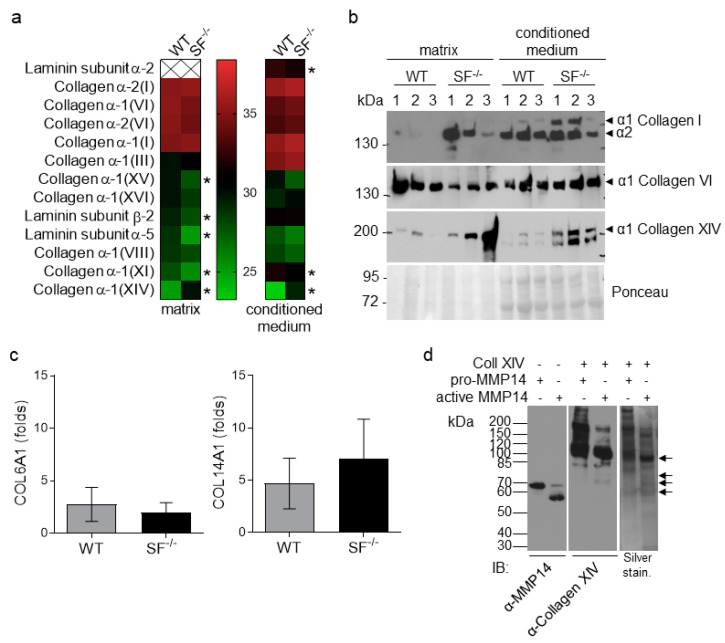
Analysis of MMP14^Sf−/−^ and MMP14^Sf+/+^ fibroblast matrices. (**a**) Mass Spectrometric and (**b**) immunoblot analysis of fibroblast matrix and conditioned medium (8% SDS-PAGE). (**c**) Transcriptional analysis of fibroblast lysates for collagens VI (COL6A1) and XIV (COL14A1). (**d**) In vitro processing of recombinant collagen XIV (Coll XIV) and human MMP14 visualized on immunoblot and silver staining (4–12% Bis-Tris gradient gel). Collagen XIV fragments generated by MMP14 are indicated with black arrows. MMP14^Sf+/+^ (WT), *n* = 3; MMP14^Sf−/−^ (SF^−/−^), *n* = 3; pro-MMP14, inactive form of MMP14; * *p* < 0.05.

We used an unbiased approach to identify the matrix molecules altered by fibroblasts without MMP14 [12]. To this end, we cultured three independent primary fibroblast populations from each genotype, as described above, and collected both the deposited matrix and supernatants. The matrix composition and the conditioned media were analyzed using mass spectrometry (Figure 2a; Table S1). Among the data obtained, we selected differentially expressed extracellular matrix proteins. At the same time, we excluded cellular proteins that may be residues from decellularization in the accumulated “matrix.” However, only five extracellular matrices were significantly altered in the fibroblast matrix and three in the conditioned medium (Figure 2a, stars). Among those, in both matrix and secreted fractions was collagen XIV. Collagen VI and I were also increased, although not significantly, only in the secreted fractions (Figure 2a). As previously observed [12], collagen I was raised in MMP14^Sf−/−^ fibroblast matrix lysates and conditioned medium by immunoblot analysis, but not collagen VI (Figure 2b). Collagen XIV was confirmed to be up-regulated by immunoblot in MMP14^Sf−/−^ fibroblast’s matrix and supernatants, as observed in the mass spectrometric analysis (Figure 2a,b).

Although MMP14 is an enzyme with pleiotropic substrates and activities, its primary function is ECM processing [27]. To understand whether the enhanced accumulation of collagen in the absence of MMP14 activity is due to de novo synthesis or impaired degradation, we analyzed transcripts in MMP14^Sf−/−^ and control fibroblasts. As observed for collagen type I [13], type VI and XIV mRNA levels were not significantly altered (Figure 2c). While lack of transcripts regulation for collagen VI agrees with the comparable levels of proteins observed in all probes, increased collagen XIV protein was not accompanied by a relative increase in transcripts (Figure 2c). This suggests that enhanced accumulation of collagen XIV results from post-translational events, possibly impaired processing. Because it is not known whether collagen XIV is a substrate for MMP14, we performed an in vitro enzymatic assay using recombinant collagen XIV and MMP14. Active MMP14 processed collagen XIV as visible from the reduction in the full-length molecule at ca. 220 kDa. It generated 65 to 80 kDa fragments, absent when MMP14 was supplied as an inactive enzyme (Figure 2d, black arrows). These data indicate that collagen XIV is a substrate of MMP14 and suggest that the increased amounts of collagen XIV in MMP14^Sf−/−^ fibroblast matrix and conditioned medium may result from impaired degradation.

### 2.3. Collagen XIV Inhibits Pro-Invasive Activities

**Figure 3:**
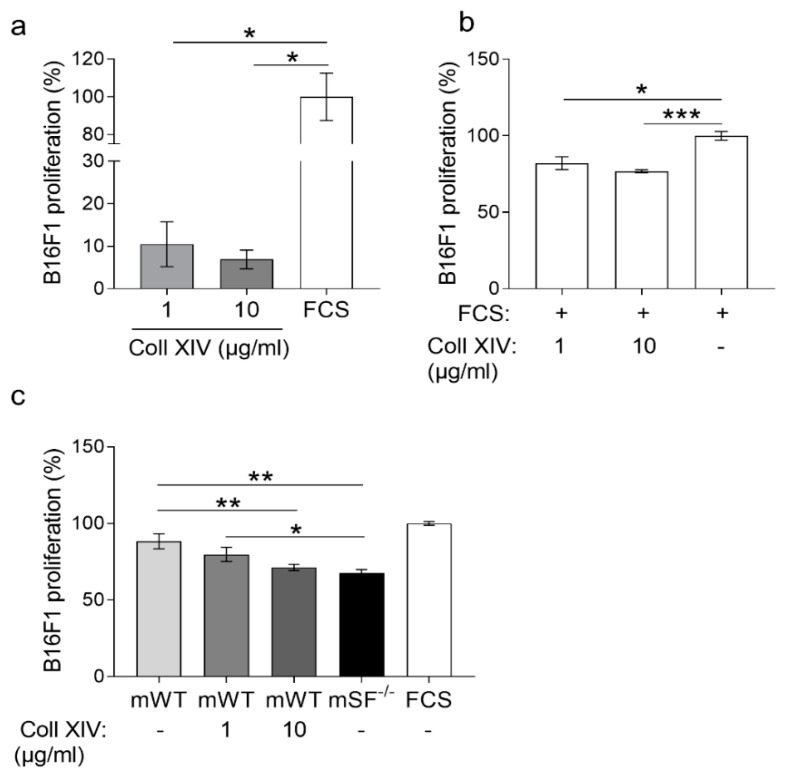
Melanoma cells proliferation on collagen XIV coatings. BrdU incorporation analysis of B16F1 cells cultured on (**a**) collagen XIV (Coll XIV) (1 to 10 µg/mL) and (**b**) on serum (FCS) alone and in combination with collagen XIV (1 to 10 µg/mL). (**c**) Proliferation was measured in cells cultured on matrix from control fibroblasts (mWT) alone or with the addition of collagen XIV (1 to 10 µg/mL) and MMP14^Sf−/−^ (SF^−/−^) fibroblasts. Experiments were repeated three times. mWT (MMP14^Sf+/+^ matrix, control matrix), *n* = 3; mSF^−/−^ (MMP14^Sf−/−^ matrix), *n* = 3; * *p* < 0.05; ** *p* < 0.01; *** *p* < 0.001.

The collagen XIV is fibril-associated collagen (FACIT) found in collagen I rich tissues, and it is known to promote fiber assembly and limit lateral growth [28,29]. Fibrotic lungs contain high collagen I and enhanced collagen XIV expression [30,31], and in liver cancer, collagen XIV contributes to maintaining the self-renewal of cancer stem cells [32]. In human skin, collagen type XIV is found underneath the sub-epidermal space and in the dermis, whereas in adult mouse skin, it is detected at low levels in the dermis [29,33]. However, little is known regarding its function in cancer and, in particular, in melanoma. To investigate the biological role of this collagen and its increase in melanoma, we used various in vitro approaches. To analyze its role in melanoma cell proliferation, we coated tissue culture plates with low (1 µg/mL) and high (10 µg/mL) concentrations of collagen XIV or FCS (positive control) and seeded melanoma cells. After 24 h, proliferation was analyzed using the BrdU incorporation method. When grown on collagen XIV, independently of the concentration, melanoma cell proliferation was low compared to FCS (Figure 3a). When collagen XIV was added to FCS, melanoma cell growth on the control substrate was reduced (Figure 3b). Thus, collagen XIV does not support proliferation and exerts an inhibitory effect when supplied to pro-proliferative substrates. This is further underscored by the experiment showing that when added to the pro-proliferative matrix deposited by control (WT) fibroblasts, collagen XIV inhibited proliferation of melanoma cells to a level comparable to that detected on matrix from MMP14^Sf−/−^ fibroblasts (mSF^−/−^, Figure 3c and Figure S2a,b). This may contribute to explaining the reduced melanoma proliferation detected on the MMP14^Sf−/−^ fibroblasts matrix, which was rich in collagen I and XIV (Figure 1c).

**Figure 4:**
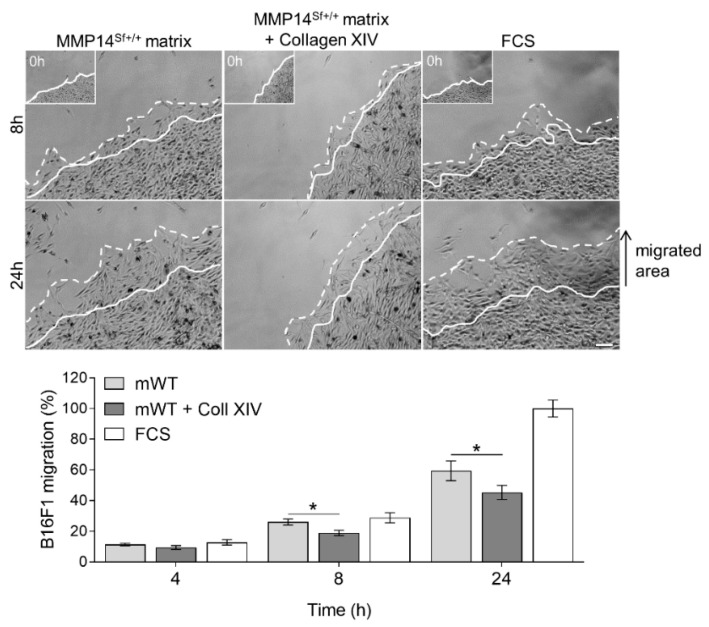
B16F1 cell migration on MMP14^Sf+/+^ fibroblast matrix enriched with collagen XIV. Colony outgrowth assay on MMP14^Sf+/+^ fibroblast matrix (mWT) containing 10 µg/mL collagen XIV (Coll XIV). The FCS-coated surface was used as a control. Below is the quantification in the percentage of the migrated area. Experiments were repeated three times. mWT (MMP14^Sf+/+^ matrix), *n* = 3; FCS, *n* = 3; a continuous line marks the cell border at time 0 h (this time point is shown in the insert); dashed line, cells border after indicated period of time; * *p* < 0.05; scale: 100 µm.

Comparably, out-migration of melanoma cells from a colony seeded on the MMP14^Sf+/+^ fibroblasts matrix supplemented with collagen XIV (10 µg/mL) was significantly reduced compared to the untreated control matrix (Figure 4).

**Figure 5:**
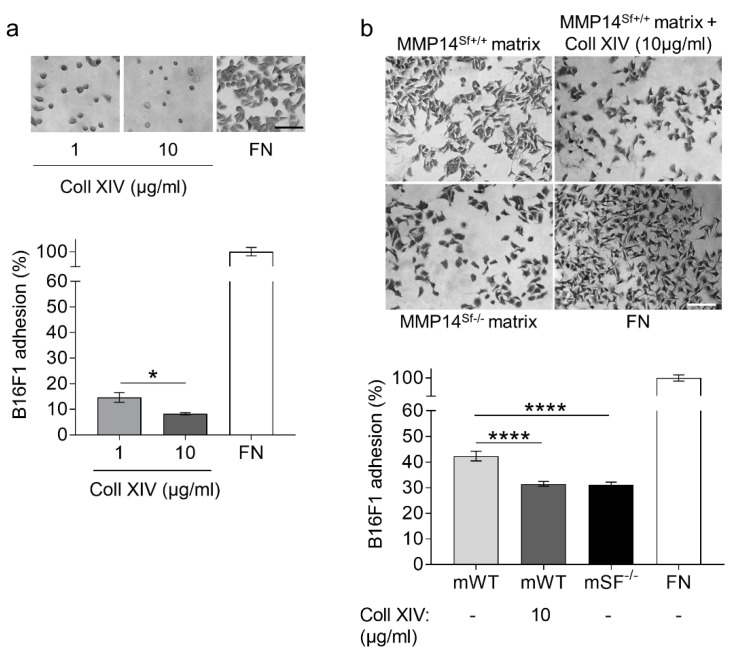
Analysis of B16F1 cell adhesion on collagen XIV coating and fibroblast matrix. (**a**) Melanoma adhesion assay on collagen XIV (Coll XIV) coatings (1 µg/mL; 10 µg/mL), (**b**) wildtype fibroblast matrix (mWT), in combination with 10 µg/mL collagen XIV (mWT + Coll XIV) and MMP14^Sf−/−^ matrix (mSF^−/−^). Fibronectin (FN) was used as positive control. Experiments were repeated three times. mWT (MMP14^Sf+/+^ matrix, control matrix), *n* = 3, mWT + Coll XIV, *n* = 3; mSF^−/−^ (MMP14^Sf−/−^ matrix), *n* = 3; * *p* < 0.05; **** *p* < 0.0001; scale, 100 µm.

The negative effect of collagen XIV in the matrix from wild-type fibroblasts during melanoma cell migration prompted us to investigate whether cells recognize and adhere well to collagen XIV. We performed an adhesion assay using collagen XIV as a substrate. Compared to the fibronectin control, the results show that cells did not adhere well to collagen XIV at a one µg/mL concentration. Surprisingly, this was significantly diminished at higher collagen concentrations (10 µg/mL) (Figure 5a). In agreement with these data, adhesion of melanoma cells on matrix deposited from MMP14^Sf+/+^ fibroblasts supplemented with collagen XIV was significantly reduced and reached levels similar to those detected on the MMP14^Sf−/−^ matrix (Figure 5b).

Collectively these data indicate that collagen XIV is not supportive of melanoma cell proliferation and migration, which are principle events of invasion.

### 2.4. Collagen XIV Is Accumulated in Melanoma Grown in Fibrotic Skin

**Figure 6:**
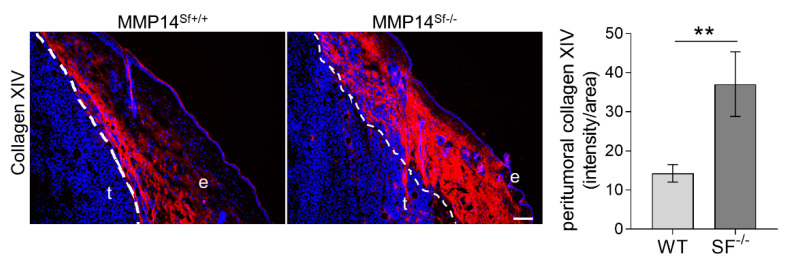
Collagen XIV expression in mouse melanoma. Immunofluorescence staining of collagen XIV (red) and DAPI (blue) in melanoma from MMP14^Sf+/+^ and MMP14^Sf−/−^ mice. The graph represents quantified signal intensity per area measured within peritumoral tissue (100 µm radius from the tumor). MMP14^Sf+/+^ (WT), *n* = 6; MMP14^Sf−/−^ (SF^−/−^), *n* = 5; t, tumor; e, epidermis. ** *p* < 0.01; scale, 100 µm.

Tumor grafting experiments with B16F1 cells, which were injected intradermally into the flank of MMP14^Sf−/−^ and control mice, showed reduced tumor growth in the absence of fibroblast-MMP14 as compared to controls [14]. The numbers of metastasis were not altered, but vascularization by blood and lymphatic vessels was decreased. Moreover, tumor cell proliferation of MMP14^Sf−/−^ mice was reduced compared to MMP14^Sf+/+^ [14]. In the peritumoral tissue of MMP14^Sf+/+^ mice, we detected a collagen XIV rich area juxtaposed to the tumor, while the remaining stroma signal intensity was lower (Figure 6). On the contrary, in tumor sections from the MMP14^Sf−/−^ mice, collagen XIV was detected intensely in peritumoral areas, and overall in the dermis (Figure 6), with some positivity extending within the melanoma (t). Those observations were confirmed by quantifying collagen XIV signal intensity in peritumoral areas (Figure 6 graph).

### 2.5. Collagen XIV Expression in Human Tissue

We showed that increased collagen I [14] and XIV negatively influence melanoma growth in mice when fibroblast-MMP14 is deleted. While the expression of collagen I in melanoma has been described [34], there are no previous studies analyzing collagen XIV expression in human melanoma. To investigate collagen XIV in melanoma, we performed immunostaining in human tissue samples of benign nevi and melanoma and further investigated MMP14 expression.

**Figure 7:**
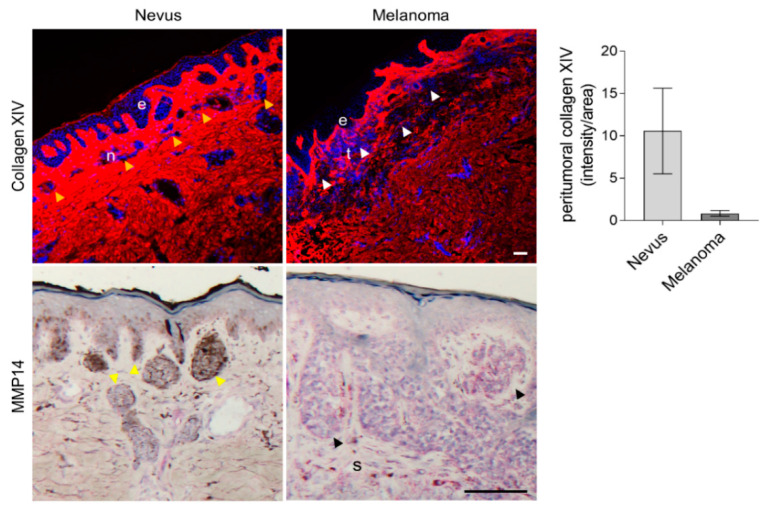
Analysis of collagen XIV and MMP14 expression in human tissue samples. Immunofluorescence detection of collagen XIV (red) and immunohistochemical staining for MMP14 in human nevi and melanoma. The nuclei are in blue (DAPI). Average collagen XIV signal intensity per area is shown on the left. Yellow arrowheads, nests of melanocytic nevi; white/black arrowheads, cutaneous melanoma nests; e, epidermis; n, nevus; t, tumor; s, stroma; scale, 100 µm.

The characteristics of the patients’ cohort used are shown in Table S2. Collagen XIV in nevi was strongly expressed throughout the dermis and was more intense and packed in areas surrounding the nests of melanocytic nevi (yellow arrowheads, Figure 7). In contrast, the peritumoral areas of melanoma showed a lower expression of collagen XIV (white arrowheads, Figure 7). Quantification of the staining intensity in tissues shows a reduction of collagen XIV expression in melanoma (Figure 7). Correlating with a direct function of MMP14 on collagen XIV processing, peritumoral areas of melanoma displayed a high expression of MMP14 (s in Figure 7) compared to stromal areas around the melanocytic nests of nevi.

Thus, the expression of collagen XIV inversely correlates to melanoma progression, and MMP14 expression in these tissues [35] hints at a suppression function for this protein in the transition from benign nevus to malignant melanoma.

## 3. Discussion

Tumor growth depends on events such as cell proliferation, migration, and invasion regulated by tumor cell-autonomous and cell-extrinsic mechanisms modulated by the interaction with the surrounding tumor microenvironment. Enhanced deposition and crosslinking of collagen, the major component of the ECM, and the resulting increased stiffness has been implicated to have a regulatory function in the progression of several tumor types [25,26]. Increased collagen density and high stiffness enhanced the proliferative state of melanoma [36]. On the contrary, several in vitro studies, including ours [14], indicate a negative role of high tissue stiffness and fibrillar collagen concentration on melanoma cell growth and migration [37,38,39,40]. In line with these findings, we also found reduced melanoma growth in vivo when collagens were increased in the dermis due to MMP14 deletion in fibroblasts or by bleomycin induction of fibrotic lesions formation [14]. However, apart from alterations in collagen I, we did not analyze whether additional modifications in the peritumoral matrix of the MMP14^Sf−/−^ also restrained melanoma growth. In vitro, we detected growth inhibition by culturing murine melanoma cells on matrices produced by MMP14-deficient fibroblasts, which macroscopically displayed enhanced matrix deposition. Although we did not find in vivo or ex vivo differences in the migratory capacity of melanoma cells in the dermis from MMP14^Sf−/−^ mice, in vitro, we detected reduced melanoma migration on matrices produced by MMP14-deficient fibroblasts. We can envision that the discrepancies between in vitro, in vivo, or ex vivo reside in the three-dimensional structure in which the cells are located in the last two systems. Thus, in vitro in a 2D environment, they rely more on integrin-based ECM adhesion and proteolysis [41,42,43]. Cells can also engage in the amoeboid migration mode in a three-dimensional network where neither integrins nor the formation of focal adhesions and proteolysis is involved [44].

Aiming to identify the different matrix compositions in the absence of fibroblasts-derived MMP14, we have used an unbiased approach and limited our search to the extracellular environment by decellularizing the sample’s previous analysis. We found in deposited or secreted proteins by fibroblasts in culture the most significant changes occurring in laminins (α-5, β-2) and collagens (XI, XV, XVI). Although collagen XI and XV were down-regulated, only XIV was strongly up-regulated in the matrix and soluble fractions. This enhancement was also detected in vivo in peritumoral areas of melanoma in MMP14^Sf−/−^ mice. Collagen XII and XIV are FACIT collagens that decorate fibrillar collagens, contribute to collagen fibrillogenesis, and are often expressed in tissues of high mechanical stress [28,33,45]. However, very little is known regarding the biological function of collagen XIV in cells and cancer in general.

Our investigations show that collagen XIV in vitro was not supportive of melanoma cells’ proliferation and could also function as an inhibitor of pro-proliferative matrix molecules. Indeed, if supplied to control fibroblast deposited matrix, collagen XIV was able to reduce the extent of melanoma cell proliferation to levels comparable to those observed on matrix from MMP14^Sf−/−^ cells that contain high amounts of this collagen. In agreement with these findings, previous studies associated collagen XIV expression mainly with differentiated tissues rather than proliferative ones [46,47] and have also shown a negative function on cellular proliferation in several other cell types [46,48,49]. Furthermore, global deletion of collagen XIV in mice led to increased cardiomyocytes proliferation, underscoring its negative role on proliferation [50].

Besides its role in proliferation, collagen XIV had a similar function on cell migration. Indeed, adding collagen XIV to the matrix deposited by control fibroblasts reduced cell migration and adhesion. Although these indicate that collagen XIV is a poor adhesive substrate, we did not find altered melanoma migration in an ex vivo invasion system [14], thus suggesting that other proteins may compensate for this in vivo.

The putative role of collagen XIV in melanoma growth is probably not limited to the mouse system. In human benign nevi, we found a strong expression of collagen XIV in areas surrounding the melanocyte nests. In contrast, this was reduced in the peritumoral stroma of malignant melanoma. In agreement with our data and the postulated negative role of collagen XIV on melanoma growth in mice, highly proliferative human melanomas show reduced collagen XIV expression in the adjacent stroma [51]. In the mouse model, the accumulation of collagen XIV can be attributed to the deletion of MMP14 in fibroblasts, leading to reduced proteolytic processing. Here we show that collagen XIV is a new substrate for MMP14. Apart from its degradative activity, it is possible to speculate, although no studies support this, that MMP14 proteolytic activity generates collagen XIV bioactive fragments supportive for melanoma growth. This issue will be a matter of future investigations.

In human tissues, we detected an inverse correlation between collagen XIV and MMP14. We found enhanced expression of collagen XIV around the melanocytic nests of benign nevi with reduced MMP14 expression, whereas around malignant melanomas, collagen XIV was low and MMP14 highly expressed. In line with these data and with a negative role of collagen XIV with melanoma progression, in human benign nevi, MMP14 is expressed low and increases with melanoma progression [35]. Importantly, MMP14 belongs to a set of up-regulated genes specifically expressed in the transition from nevi to non-metastatic and metastatic melanoma [52].

In summary, we show that the fibroblast-specific deletion of MMP14 leads to changes in the peritumoral ECM composition modulating melanoma growth. Enhanced accumulation of collagen XIV in the MMP14^Sf−/−^ fibroblast matrix does not support cell proliferation and migration, thus reducing melanoma cell proliferation, migration, and adhesion to the fibroblast matrix. Moreover, we identified collagen XIV as a substrate for MMP14, showing that enhanced collagen XIV correlates with reduced melanoma growth in mice. In support of the negative role of collagen XIV on malignancy, in human samples, collagen XIV is decreased upon transition from benign nevus to invasive melanoma.

## 4. Materials and Methods

### 4.1. Cell Culture

B16F1, B16F0 [53], and HCmel12 [54] cells were grown in DMEM medium (+4.5 g/L D-Glucose, L-Glutamine, Thermo Fisher Scientific, Darmstadt, Germany), supplemented with 10% FCS, 2 mM L-glutamine, 100 U/mL penicillin, and 100 µg/mL streptomycin. Primary mouse dermal fibroblasts were isolated and cultured as previously described [13]. Cells were routinely tested for mycoplasma (PCR Mycoplasma Test Kit I/C; PK-CA91-1096; Promocell, Heidelberg, Germany). Fibroblast conditioned medium was prepared by culturing primary fibroblasts in serum-free DMEM for 24 h. The medium was collected and spun down for 5 min at 315× *g* to remove cellular debris. A fibroblast matrix was prepared by culturing primary fibroblasts in complete DMEM with 0.05 g/L L-ascorbic acid (Sigma, Taufkirchen, Germany) for 14 days; the medium was changed every 2–3 days. Fibroblasts were removed from the matrix by adding 20 mM NH4OH and 0.5% Triton X-100. The matrix was washed gently with PBS and either collected for further analysis or visualized by staining with 0.25% Coomassie Brilliant blue (G250, Sigma, Taufkirchen, Germany). Images were recorded using a light microscope equipped with DISKUS 4.50.1638 software. Recombinant collagen XIV was produced as previously described [33]. Wildtype fibroblast matrices and multi-well plates were coated with recombinant collagen XIV (1 µg/mL; 10 µg/mL) diluted in PBS, 100% FCS alone, or in combination with collagen XIV (1 µg/mL; 10 µg/mL) o/n (overnight) at 4 °C. Potential nonspecific binding sites were blocked with 1% heat-inactivated BSA for one hour at RT.

### 4.2. Cell Migration: Colony Outgrow Assay

B16F1 cell migration assays were performed as previously described [55]. Briefly, B16F1 cells were treated with 1.6 µg/mL mitomycin-C (M4287, Sigma-Aldrich, Schnelldorf, Germany) in 0.1% FCS DMEM for 4 h at 37 °C to inhibit cell growth. Cells (2 × 10^5^ cells/ring) were seeded on top of the fibroblast matrix, restricted by cloning rings (0.5 mm diameter) and left to adhere for three h. After removing the ring, cells were washed with PBS to remove non-adherent cells, and a serum-free medium was added. Cell migration was monitored on an Olympus XM10 microscope (Olympus, Tokyo, Japan) and evaluated with ImageJ software (http://rsb.info.nih.gov/ij; version ImageJ 1.53a, accessed on 1 January 2017).

### 4.3. BrdU Incorporation Assay

Melanoma cell proliferation assays were performed as previously described [14]. Briefly, cells were starved for 24 h and seeded on pre-coated wells for 24 h. Cellular proliferation was analyzed using the Cell Proliferation ELISA^®^ Kit (Cell Proliferation ELISA, BrdU kit, Cat. No. 11 647 229 001, Roche Life Science, Penzberg, Germany) according to the manufacturer’s instructions.

### 4.4. In Vitro Cleavage Assay

Recombinant human MMP14 100 ng/µL (918-MPN, R&D Systems, Wiesbaden, Germany) was activated using 0.1 µg/mL trypsin (15090046, Thermo Fisher Scientific, Darmstadt, Germany) in 50 mM Tris, 0.15 M NaCl, 10 mM CaCl_2_, 5 µM ZnCl_2_, 0.05% Brij-35, pH 7.5 for 1 h at 37 °C. Trypsin was inactivated by adding 1 mM pefablock for 15 min at RT (31682, Serva, Heidelberg, Germany). Afterward, active MMP14 was diluted in assay buffer (50 mM Tris, 3 mM CaCl_2_, 1 µM ZnCl_2_, pH 8.5) to a final concentration of 50 ng/µL. Active MMP14 (100 ng) was used to cleave 1 µg recombinant collagen XIV in assay buffer for 2 h at 37 °C. Cleavage was confirmed by immunoblot and silver gel staining (4–12% Bis-Tris gradient gel) (24612, Thermo Scientific, Asbach, Germany). Proteolytic fragments were further identified by peptide mass fingerprint analysis at the CECAD/CMMC Proteomics Facility at the University of Cologne.

### 4.5. Immunoblot

The fibroblast matrix was solubilized in 8 M Urea, 0.2% SDS, 0.5 M TEAB, 5 mM TCEP, protease inhibitor, and analyzed as previously described [14]. The nitrocellulose membrane was incubated with the following primary antibodies: rabbit α-collagen I, 1:500, (ab21286, Abcam, Cambridge, UK); rabbit anti-collagen VI, a kind gift of Raimund Wagener (Institute for Biochemistry II, Cologne, Germany); rabbit α-collagen XIV [33]; and rabbit anti-MMP14 [10]. Membranes were washed three times with PBST for 5 min and incubated for 1 h at RT with the HRP-conjugated secondary antibody: swine α-rabbit (P0217, Dako, Waldbronn, Germany).

### 4.6. Preparation of Samples for Proteome Analysis

Samples for proteome analysis were precipitated with acetone and solved in 8 M urea. Afterward, samples were reduced with 5mM dithiothreitol (DTT) for 1 h at 37 °C, alkylated with 40mM chloroacetamide (CAA) for 30 min RT, and digested with endopeptidase lys-C for 4 h at 37 °C. Urea concentration was brought to 2M with triethylammonium bicarbonate (TEAB), and samples were digested overnight with trypsin at a 1:75 ratio (*w*/*w*). Samples were acidified with 1% formic acid. Salts and impurities were removed by micro-purification with stage tips containing 2 layers of SDP-RPS (poly(styrene-divinylbenzene)-reversed-phase sulfonate) [56]. Samples were analyzed with a Q-Exactive Plus (Thermo Scientific, Asbach, Germany) mass spectrometer coupled to an EASY nLC 1200 UPLC (Thermo Scientific, Asbach, Germany) at the CECAD/CMMC Proteomics Facility at the University of Cologne, as described earlier [57].

### 4.7. Tumor Grafting Experiments

Tumor grafting experiments were performed as previously [14]. Briefly, B16F1 melanoma cells (0.5 × 10^6^ cells/animal) were injected intradermally into the flank of BL6 MMP14^Sf+/+,^ and MMP14^Sf−/−^ mice, and tumor growth was followed over time. The experiment was terminated, and mice were euthanized after tumor sizes reached the maximal allowed tumor size.

### 4.8. Immunofluorescence Staining

Cryosections of mouse melanomas and human tissues were immunostained as previously described [14]. Briefly, cryosections were fixed and permeabilized with ice-cold acetone for 2 min, dried, and blocked with 10% NGS for 30 min at RT. Primary antibody diluted in 1% BSA in PBS was added to the sections and incubated in a humidified chamber overnight at 4 °C. Sections were washed three times with PBS and incubated in secondary antibody (goat anti-rabbit Alexa-594 nm, A11037, Thermo Fisher Scientific, Darmstadt, Germany) in 1% BSA in PBS in a humidified chamber for 1 h at RT. Signal intensities were quantified with ImageJ software. Primary antibodies specific for human (KR71) and mouse (KR47) collagen XIV were produced as described previously [29].

### 4.9. Immunohistochemical Staining

Immunohistochemistry was performed using human melanoma sections. Sections were fixed with ice-cold acetone for 8 min, blocked with 5% BSA in 0.05% TBS-Tween for 1 h, and incubated overnight with the primary antibody to MMP14 (Chemicon, MAB3328, 1:200, Nürnberg, Germany). Secondary antibodies were detected using the DCS detection Line (DCS Innovative Diagnostic Systems, AD000RP, Hamburg, Germany) and Fast Red as substrate (XBioGenex, HK182-5KE, Fremont, CA, USA).

### 4.10. RNA Isolation, RT-PCR, and Real-Time PCR

RNA was isolated from primary fibroblast cells, lysed with RNAzolTM B (WAK-CS-105, WAK-Chemie, Taunus, Germany), and incubated on ice for 5 min. Cells were scratched and inverted with phenol-chloroform (X085.1, Roth, Karlsruhe, Germany) for 15 s. After incubation on ice for 3 min, samples were centrifuged at 16,000× *g* for 15 min at 4 °C, and RNA was precipitated by adding isopropanol to the supernatant. Samples were incubated overnight at −80 °C and centrifuged for 10 min at 16,000× *g* at 4 °C. The pellet was washed with 70% EtOH and resuspended in H^2^O. Reverse transcription and quantitative real-time PCR were performed as described previously [14]. Primers for the amplification of COL6A1 and S26 have been previously described [10,58]. Primers for COL14A1 were for 5′ TGGTGGAGAGCCTGACCCGG 3′; rev 5′ GCATCCCACCTGACGCGCAT 3′; accession number: BC138345.

### 4.11. Statistics

Statistical analysis was performed using GraphPad Prism version 7.05 for Windows (GraphPad Software, La Jolla, CA, USA, www.graphpad.com). The student’s *t*-test was performed for data analysis with *p* < 0.5 considered as statistically significant

## Data Availability

The data presented in this study are available on request from the corresponding author. The data are not publicly available due to privacy.

## Supplementary Materials

The following are available online at www.mdpi.com/article/10.3390/ijms222212276/s1.
