# Supplementary material for: Extracellular Matrix Remodeling by Fibroblast-MMP14 Regulates Melanoma Growth
Source: Int J Mol Sci. 2021 Nov 12;22(22):12276. doi: 10.3390/ijms222212276 (PMC8625044; doi:10.3390/ijms222212276)
Supplement: Supplementary file 1 [file ijms-22-12276-s001.zip › ijms-1422063-supplementary.pdf]

International Journal of
Molecular Sciences
MDPI

Supplementary Materials

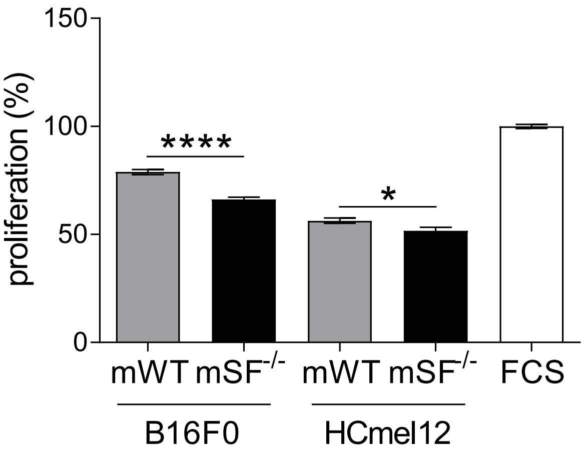

Figure S1. Analysis of melanoma proliferation on fibroblast deposited matrix. BrdU incorporation measurement of B16F0 and HCmel12 cells cultured on fibroblast matrix from MMP14$^{Sf+/+}$ (mWT, n=3) and MMP14$^{Sf-/-}$ (mSF$^{-/-}$, n=3). Experiments were repeated twice. *p< 0.05; ****p<0.0001.

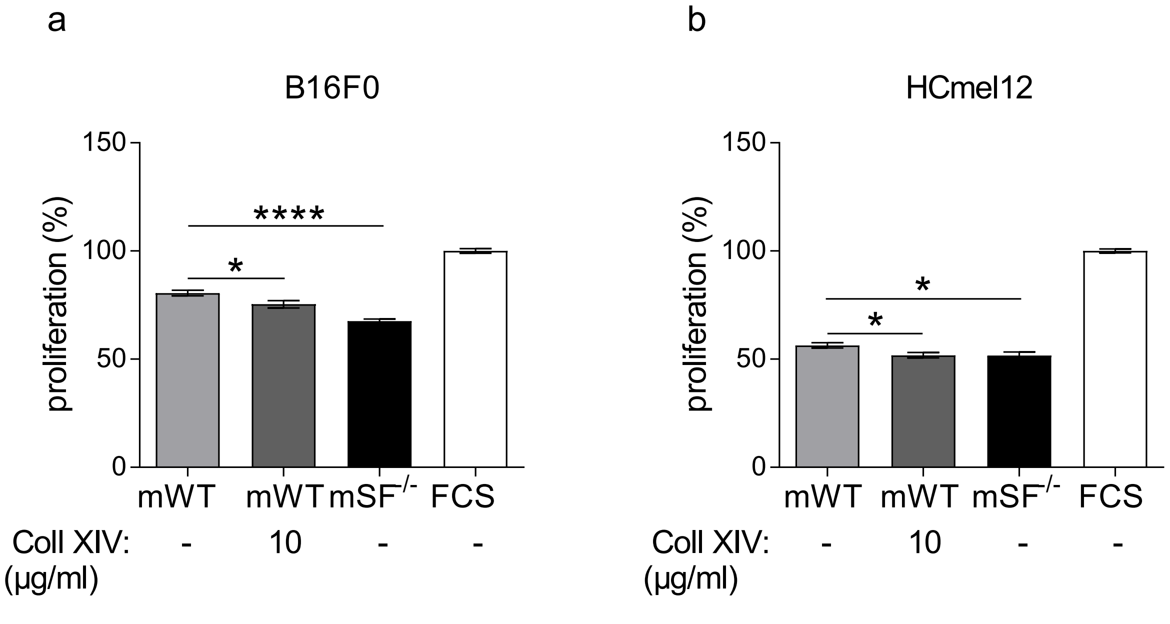

Figure S2. Melanoma cell proliferation on fibroblast matrices in combination with collagen XIV. Proliferation of B16F0 (a) and HCmel12 (b) cells was measured after culturing on matrix from control fibroblasts (mWT) alone or with the addition of collagen XIV (10µg/ml), and MMP14$^{Sf-/-}$ (SF$^{-/-}$) fibroblasts. *p< 0.05; ****p<0.0001.

Table S1. Mass Spectrometry data from selected extracellular matrix proteins of MMP14$^{Sf+/+}$ (WT) and MMP14$^{Sf-/-}$ (SF$^{-/-}$) fibroblast and conditioned media. Represented are the averaged data of three independent samples for MMP14$^{Sf+/+}$ (WT) and MMP14$^{Sf-/-}$ (SF$^{-/-}$). n.d., not detected.

	**matrix**			**conditioned medium**		
**Protein name**	**WT mean**	**SF$^{-/-}$ mean**	**p-value**	**WT mean**	**SF$^{-/-}$ mean**	**p-value**
Laminin subunit α-2	-	-	-	31,8	31,0	0,03
Collagen α-2(I)	35,3	35,2	0,89	35,2	35,3	0,91
Collagen α-1(VI)	35,7	35,1	0,46	32,9	33,7	0,07
Collagen α-2(VI)	35,5	34,8	0,44	32,4	33,1	0,09
Collagen α-1(I)	34,8	34,9	0,66	35,0	35,3	0,63
Collagen α-1(III)	30,1	30,5	0,53	34,2	34,3	0,94
Collagen α-1(XV)	29,9	26,6	0,05	28,7	26,0	0,08
Collagen α-1(XVI)	29,7	28,2	0,26	28,9	28,8	0,94
Laminin subunit β-2	29,6	27,9	0,03	30,6	30,2	0,52
Laminin subunit α-5	28,4	25,0	0,02	26,7	25,2	0,24
Collagen α-1(VIII)	28,8	28,4	0,58	27,6	27,1	0,21
Collagen α-1(XI)	28,1	25,3	0,00	31,3	29,6	0,04
Collagen α-1(XIV)	25,6	31,4	0,03	23,2	29,8	0,01

Table S2. Data relative to the used human tissues. Nevi n=9; melanoma n=16; TD, tumor depth.

	**Sex**	**Site**	**TD (mm)**	**morphology**
**Nevi**				
	M	Trunk	-	congenital
	F	Extremities	-	congenital
	F	Extremities	-	-
	F	Extremities	-	-
	M	Extremities	-	compound
	M	Extremities	-	dermal
	F	Trunk	-	dermal
	M	Head	-	congenital, compound
	W	Head	-	blue nevus
**Melanoma**				
	M	trunk	2,9	
	M	extremities	2,8	
	M	trunk	2	
	M	trunk	1,1	
	W	trunk	0,4	
	W	extremities	0,75	
	F	extremities	10,8	
	F	trunk	18,7	
	F	extremities	10	
	M	extremities	4,5	
	M	head	9	
	M	trunk	5,8	
	W	extremities	12	
	W	head	7,3	
	M	trunk	9,6	
	W	extremities	4,4	
